# 2144. A Phase 1, Multicenter, Open-Label, Parallel-Group Study to Assess the Safety and Pharmacokinetics (PK) of Oral Epetraborole Tablets in Adult Subjects with Varying Degrees of Renal Function

**DOI:** 10.1093/ofid/ofad500.1767

**Published:** 2023-11-27

**Authors:** Gina Patel, George C Canas, Kimberly Cruz, Thomas C Marbury, Mark Gotfried, Wallace (Boon Chun) Chan, Kenneth Duchin, Gary Maier, Sanjay Chanda, Linda Kammerer, Jennifer Long, Stephanie Moore, Kevin O’Shea, Afshin Shafiee, Alex Smith, Paul B Eckburg

**Affiliations:** Patel Kwan Consultancy LLC, Madison, Wisconsin; Kidney Specialists of Minnesota, Brooklyn Center, Minnesota; Advanced Pharma CR, LLC, Miami, Florida; Orlando Clinical Research Center, Orlando, FL; University of Arizona, Phoenix, Phoenix, Arizona; Patel Kwan Consultancy, Madison, Wisconsin; Patel Kwan Consultancy, Madison, Wisconsin; Maier Metrics and Associates, LLC, Worcester, Massachusetts; AN2 Therapeutics, Inc., Menlo Park, California; AN2 Therapeutics, Menlo Park, California; AN2 Therapeutics, Inc., Menlo Park, California; AN2 Therapeutics, Menlo Park, California; AN2 Therapeutics, Menlo Park, California; AN2 Therapeutics, Menlo Park, California; AN2 Therapeutics, Menlo Park, California; AN2 Therapeutics, Menlo Park, California

## Abstract

**Background:**

Epetraborole (EBO), an orally available bacterial leucyl transfer RNA synthetase inhibitor with potent activity against nontuberculous mycobacteria, is in clinical development for treatment-refractory MAC lung disease. The objective of the study was to evaluate EBO PK in adult subjects with varying degrees of renal impairment (RI) including end stage renal disease (ESRD) with intermittent hemodialysis (IHD).

**Methods:**

Open-label, single-dose EBO 500 mg PO was given to subjects in 5 cohorts: normal renal function, mild, moderate, and severe RI, and ESRD-IHD (Table); ESRD subjects received a second 500 mg dose on Day 5 approximately 1 hour before receiving IHD. EBO and its inactive major metabolite (M3) concentrations in plasma, urine and dialysate were measured by validated LC-MS/MS methods. Plasma PK parameters were determined using non-compartmental methods and compared among cohorts using analysis of variance (ANOVA). Standard clinical and laboratory evaluations and treatment-emergent adverse events (TEAEs) were assessed.

**Results:**

40 subjects were enrolled (8/cohort). Subjects with RI did not exhibit quantitatively distinct EBO plasma PK profiles compared to those with normal renal function; AUC mean ratios were 110-140% in subjects with RI, and the mean ratios of maximum observed concentration (C_max_ ) values did not exceed 116% (Table). The elimination half-life (t_1/2_) increased slightly from 9.3 to 11.0 h in ESRD subjects, and clearance decreased by about 30%. Renal elimination was not a major route of excretion (∼15% of dose over 72 h) for EBO, with mean renal clearance ranging from 4.24 L/h to 1.04 L/h. Metabolite M3 AUC increased 4-fold in subjects with severe RI, and t_1/2_ increased from 20 to 32 h. EBO was well tolerated; 7 subjects (17.5%) experienced at least 1 TEAE (11 events), all mild in severity except 1 moderately-severe TEAE of worsening anemia. There were no severe or serious TEAEs.

**Figure:**
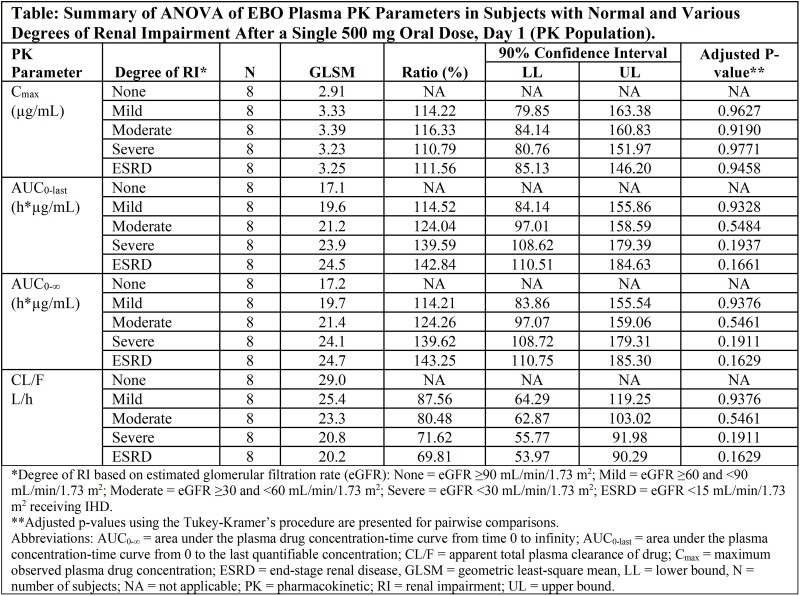

**Conclusion:**

Minimal increases in plasma EBO exposures and similar t_1/2_ values were observed in subjects with varying degrees of RI, including ESRD-IHD. Single 500 mg doses of EBO were well tolerated in each RI cohort. Overall, these data suggest that no dose adjustment of EBO is needed in subjects with any degree of RI.

**Disclosures:**

**All Authors**: No reported disclosures

